# Immune dysregulation in tuberculosis-diabetes comorbidity: mechanistic and translational insights

**DOI:** 10.3389/fimmu.2026.1803046

**Published:** 2026-04-23

**Authors:** Aminat Y. Saula, Muge Cevik, Jacqueline M. Cliff, Katharina Ronacher, Ruth Bowness

**Affiliations:** 1 Centre for Mathematical Biology, Department of Mathematical Sciences, University of Bath, Claverton Down, Bath, United Kingdom; 2 Division of Infection and Global Health Research, School of Medicine, University of St Andrews, St Andrews, Scotland, United Kingdom; 3 Centre of Inflammation Research and Translational Medicine, and Department of Biosciences, Brunel University of London, London, United Kingdom; 4 Translational Research Institute, Mater Research Institute, The University of Queensland, Brisbane, QLD, Australia; 5 Australian Infectious Diseases Research Centre, The University of Queensland, Brisbane, QLD, Australia

**Keywords:** diabetes mellitus, immune response, immunity, tuberculosis, immune dysregulation, comorbidity

## Abstract

**Background:**

Tuberculosis (TB) remains a leading cause of infectious disease mortality worldwide, and the rising prevalence of diabetes mellitus (DM) represents a major obstacle to TB control. DM increases susceptibility to TB, worsens disease severity, delays treatment response, and is associated with poorer outcomes, largely through disruption of host immunity.

**Methods:**

We conducted a systematic review of studies published between 1974 and May 31, 2023 that examined immunological mechanisms through which DM alters TB pathogenesis. In total, 81 eligible studies involving animal models, human participants, or combined approaches were identified and synthesised across different stages of TB.

**Results:**

Across studies, DM was associated with broad dysregulation of innate and adaptive immune responses, altered cytokine signalling, impaired granuloma structure and function, and reduced control of *Mycobacterium tuberculosis (Mtb)*. Distinct immune profiles emerged between TB disease with DM and latent TB infection with DM, with heterogeneity partly explained by differences in study design, metabolic status, and disease stage. Importantly, emerging evidence indicates that pre-diabetes and intermediate hyperglycaemia may also compromise TB immunity and contribute to disease progression.

**Conclusion:**

Our findings highlight DM as a key immunometabolic modifier of TB pathogenesis. They also suggest that earlier metabolic optimisation and host-directed therapeutic strategies could be explored as potential approaches to improve outcomes in this growing high-risk TB-DM population.

**Systematic review registration:**

https://www.crd.york.ac.uk/PROSPERO/, identifier CRD42023431040.

## 1 Introduction

In 2022, the World Health Organization (WHO) identified tuberculosis (TB) as the second deadliest infectious disease globally, following COVID-19 and ahead of HIV/AIDS (1). By 2023, TB had become the leading infectious cause of death, affecting an estimated 10.8 million people, with *Mycobacterium tuberculosis* (*Mtb*) typically infecting the lungs (2). In the 2025 WHO Global Tuberculosis Report, a light reduction compared to the 2023 estimate was reported, with 10.7 million TB cases in 2024, still above the pre-pandemic estimated 10.3 million cases in 2020 (3). Tuberculosis infection (TBI) differs from TB disease: TBI is marked by a sustained immune response to *Mtb* antigens without clinical signs, whereas TB disease involves symptoms and higher bacterial loads (4–6). The term TBI has replaced latent tuberculosis infection (LTBI), acknowledging that this group includes both protected individuals and those progressing toward disease. This distinction may explain variability in study findings. Around 10% of TBI cases progress to TB disease, which, if untreated, has a mortality rate exceeding 50% (6–8).

Typically, in response to *Mtb*, the immune system forms granulomas to contain the bacteria, though these can also serve as reservoirs for *Mtb* (9). In immunocompetent hosts, coordinated cytokine and chemokine secretion supports effective immune responses (10, 11). These responses are often broadly categorised into functional types. Type 1 (Th1) responses promote macrophage activation and are essential for controlling intracellular pathogens such as *Mtb*, type 2 (Th2) responses are more regulatory and support tissue repair, and type 17 (Th17) responses drive neutrophil recruitment and inflammation (10, 12–15). Further aspects of TB immunology have been addressed in detail by Dheda et al. (16).

The WHO now recognises DM as one of the major risk factors for new TB cases, alongside HIV infection, undernutrition, smoking, and alcohol use disorders (3). The growing burden of diabetes mellitus (DM) complicates TB outcomes, as DM impairs immunity, increasing TB risk and severity (17–20). Global DM prevalence rose from 108 million in 1980 to 451 million in 2017, with projections of 693 million by 2045 (21). Diabetic individuals face a threefold increased risk of developing TB disease (22, 23), with higher bacterial loads (24), delayed sputum culture conversion (25), increased mortality (26, 27), higher relapse (28), and elevated drug resistance-even during first TB episodes (29).

Animal and human studies show that DM comorbidity impairs pathogen defence, delaying or weakening immune responses and promoting dissemination of infection (30–32). This dysfunction involves altered cytokine and chemokine levels, shifts in immune cell populations, apoptosis, and fibrosis (33, 34). Consequently, *Mtb* clearance is delayed by both innate and adaptive immune dysregulation in DM.

Despite substantial evidence linking DM and TB, the immunopathological mechanisms underlying this association remain inadequately defined. Heterogeneity in study designs, populations, and immunological assessments has contributed to inconsistent findings. Previous reviews have examined TB-DM interface from different perspectives, including screening and epidemiology, clinical care, policy, and management, the broader pathophysiology linking tuberculosis-associated inflammation to insulin resistance and type 2 DM, and clinical outcomes in TB-DM (35–37). Our review complements these clinically and epidemiologically focused reviews by providing a systematic, comparator-based synthesis of host immune responses in TB-DM. In this systematic review, we aimed to characterise how type 2 DM alters immune responses to TB in humans, and how diabetes, modelled predominantly as type 2-like states in rodents, with additional insulin-deficient models including Ins2Akita mice and a Komeda diabetes-prone rat model of type 1 DM, affects TB immunity in experimental animals. By synthesising human and animal data, we sought to identify consistent immunological alterations that may inform targeted prevention strategies and potential therapeutic interventions for this vulnerable population.

## 2 Methods

### 2.1 Search strategy and selection criteria

The screening, selection, and data extraction processes strictly adhered to systematic review protocols. We retrieved research articles in English that report on immune responses in individuals with TBI-DM or TB-DM, including studies on TB infection in animals. Articles were primarily retrieved from MEDLINE (1946 to May 31 2023) and EMBASE (1974 to May 31 2023) databases. There is no restriction on age groups and genders. The comparator for this review comprises individuals with TB but without DM. Exclusion criteria include duplicate studies, citations without abstracts, anonymous reports, editorial or author commentaries, conference papers with insufficient immunological data, reports with inappropriate comparator groups, reports focusing on just the prevalence and incidence rate of TB-DM, TB-DM alongside other diseases/habits (e.g., multimorbidity such as HIV, smoking), and systematic reviews and meta-analyses that do not facilitate comparisons of immunological or metabolic dysfunctional responses in TB-DM patients. During the study selection process, we first screened titles and abstracts, then reviewed full-text articles, assessing them for eligibility and applying the exclusion criteria mentioned above. Full search strategy is explained in the detailed protocol for this systematic review which has been registered with PROSPERO CRD42023431040.

### 2.2 Data extraction

Two authors (AYS and MC) initially screened and retrieved articles based on the eligibility criteria. Three reviewers (AYS, MC, and RB) then evaluated the full-text articles to select those for inclusion in the study. From each selected study, the following variables were extracted: article title, first author’s last name, publication year, study setting, design, country, type of model, type of experiment, study aim, number of participants, samples collected, type of comorbidity, diagnosis of DM and TB, treatment regimen (if any), antigens and stimulation, viable *Mtb* colonies, and a list of assessed cytokines, chemokines, cytotoxic markers, immune markers, and metabolic factors.

### 2.3 Data analysis and reporting framework

Given the heterogeneity in study designs, populations, outcome measures, and reporting formats among the included studies, a meta-analysis was not feasible. Therefore, a narrative synthesis approach was employed to summarise and interpret the findings. The narrative reporting followed the general principles recommended by the Cochrane Handbook for Systematic Reviews of Interventions, including (1): grouping studies according to key themes such as study design, population characteristics, and outcomes (2); summarising the direction and strength of evidence for each outcome; and (3) identifying patterns, consistencies, and differences across studies. Where appropriate, results were contextualised in relation to methodological quality and study limitations. This approach allowed for a structured and transparent presentation of findings despite the diversity of included data.

### 2.4 Role of funders

No funding received for this study.

## 3 Results

**Figure 1:**
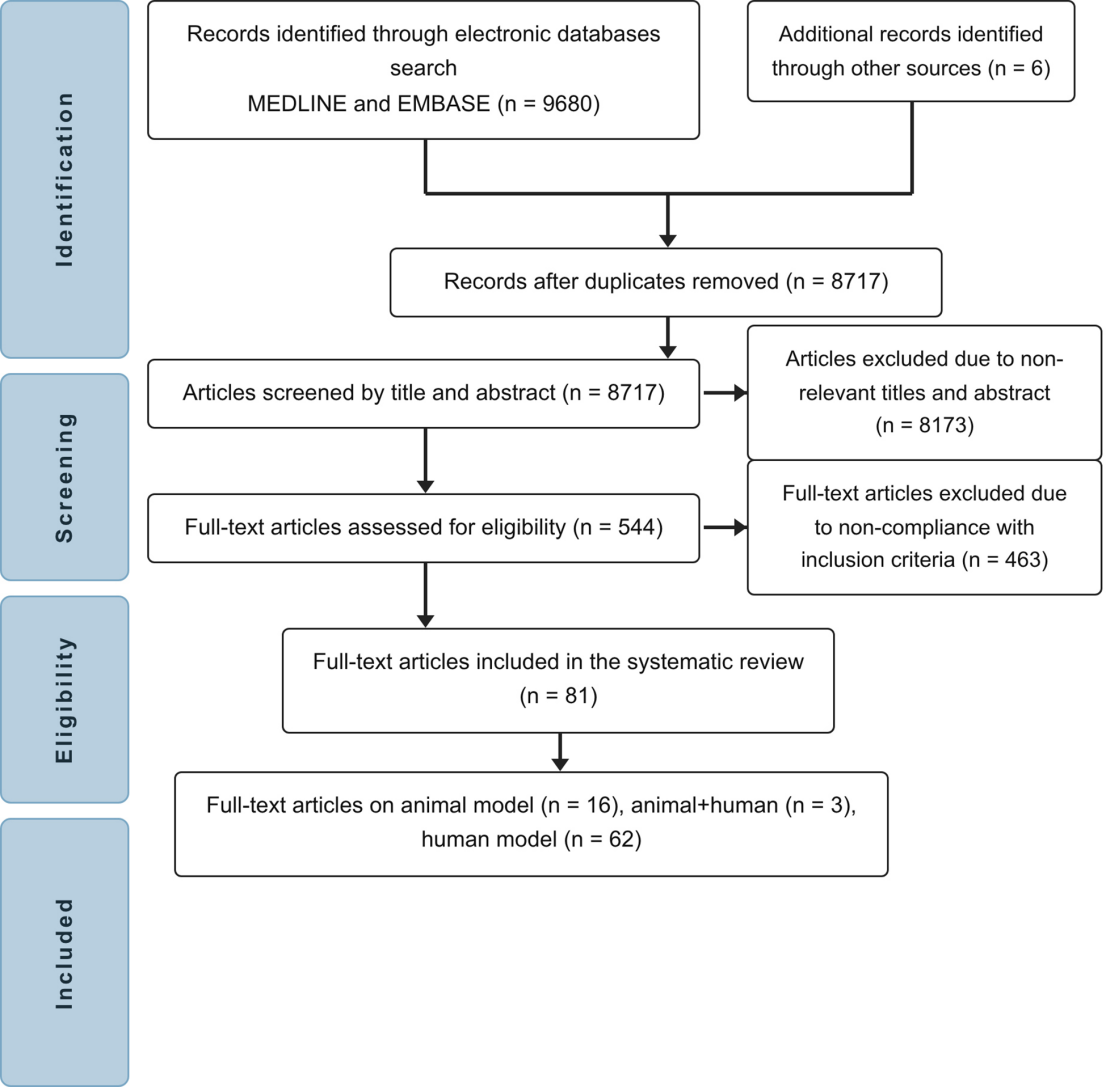
Flow chart of study selection.

Our systematic search identified 9,686 potentially relevant articles. Of these, 544 were retrieved for full-text review. After applying the eligibility criteria, a total of 81 studies were included: 16 involving animal models (comprising 120 guinea pigs, 506 mice, and an unspecified number of rats) (30, 38–54), 3 combining both animal and human studies (involving 122 mice and 106 human participants) (55–57), and 62 studies conducted exclusively in humans (involving 8,567 participants) (31, 32, 54, 58–116). Study (115) was included in accordance with the systematic review methodology applied at the time of the search, and its subsequent retraction does not affect our main conclusions. A flow chart outlining the study selection process is presented in **Figure 1**.

### 3.1 Human studies

#### 3.1.1 TBI with coincident DM

**Supplementary Table 1** details study conditions for TBI-DM comorbidity, including first author, age, gender, sample size, sample type, methodology, stimulation conditions, and any DM medications used. All studies either confirmed participants were naive to TB treatment or did not report TB treatment status.

##### 3.1.1.1 Serum and plasma cytokine concentrations in TBI-DM

TBI-DM individuals show reduced plasma levels of type 1 (IFN-γ, TNF, IL-2) and type 17 (IL-17F) cytokines (58), with similar reductions observed in whole blood (90). Most IL-20 subfamily cytokines (IL-19, IL-20, IL-24, IL-10) are also lower in TBI-DM, while IL-22 is elevated and correlates with HbA1c (65). Serum IL-38 is reduced (66), whereas TNF, IL-6, and IL-1β remain unchanged (66)—though IL-1β is elevated in newly diagnosed DM, and controlled DM shows impaired IFN-γ and reduced T-bet (103).

##### 3.1.1.2 Cytokine response to stimulation in TBI-DM

Whole blood stimulation with *Mtb* antigens in TBI-DM individuals shows reduced cytokines (IFN-γ, TNF, IL-17A, IL-10) compared to non-diabetics (58, 90). While IL-1Ra is secreted in response to *Mtb* in those with normal glucose tolerance, pre-DM, or newly diagnosed DM, it is absent in pre-existing DM patients on treatment (105).

PBMC studies report mixed results: elevated glycaemia in treated TBI-DM is linked to reduced IFN-γ and TNF after *M. bovis* BCG stimulation (83), while some *Mtb*-stimulated PBMC studies show increased Th1 and Th17 cytokines from iNKT and MAIT cells (60, 89), but reduced expression from *γδ* T cells (82) and diminished IL-13/IL-22 after PMA/Ionomycin (74). Elevated IFN-γ responses to TB antigens in TBI-DM versus euglycemic TB suggest immune profiles resembling active TB (70). These discrepancies may stem from differences in antigen type, stimulation conditions, cytokine-expressing cell subsets, analytical methods (e.g., flow cytometry, ELISA, RNAseq), birthplace, TST status, and DM medication effects (60, 70, 82, 89, 103).

##### 3.1.1.3 Cytotoxic and immune marker expressions in TBI-DM

Following PBMC stimulation with *Mtb* antigens, TBI-DM individuals showed reduced expression of cytotoxic markers perforin (PFN) and granzyme B (GZEB) compared to non-DM (60, 82, 89). However, one study found increased frequencies of CD8+ T cells expressing these markers after whole blood stimulation in TBI-DM versus non-DM (67). Despite this, all agree cytotoxic marker expression did not differ between TBI-DM and TBI-only after PMA/ionomycin stimulation. Variations likely reflect factors influencing cytokine discrepancies (60, 67, 82, 89).

Reduced immune markers CD69 and PD-1 were also seen in *Mtb*-stimulated PBMCs (82, 89), while *Mtb*-stimulated monocyte-derived macrophages (MDM) from DM patients showed decreased HLA-DR, CD80, CD86 and increased PD-L1, especially with high-virulence strains (93).

##### 3.1.1.4 Innate and adaptive immune cell populations in TBI-DM

TBI-DM is linked to reduced frequencies of innate lymphoid cells (ILC2, ILC3) compared to TBI alone (74), along with lower NK37 and *γδ* T cells (82), but increased iNKT (89) and MAIT (60) cells. Effector memory CD4+ T cells, naive B cells, myeloid and plasmacytoid dendritic cells (DCs), and classical/intermediate monocytes are also reduced in TBI-DM, while activator/atypical B cells and non-classical monocytes are elevated (78). Additionally, TBI-DM shows compromised CD4+ T cell responses with reduced Th1, Th2, and Th17 frequencies, linked to DM-modulated IL-10 and TGF-β signaling (90).

##### 3.1.1.5 Metabolic profiles in TBI-DM

Metabolic changes in TBI-DM include reduced systemic adipokines like adiponectin and adipsin (72), potentially impacting inflammation regulation (117) and adipose tissue stability (118). Conversely, leptin, visfatin, and PAI-1 levels are elevated in TBI-DM versus non-DM individuals (72).

#### 3.1.2 TB with coincident DM

**Supplementary Table 2** details studies on TB-DM comorbidity included in this review, covering individuals on antituberculosis treatment and comparisons by treatment duration.

##### 3.1.2.1 Serum, plasma, and BALF cytokine concentrations in TB-DM

Studies show TB-DM individuals have elevated serum pro-inflammatory cytokines-TNF, IFN-γ, IL-2, IL-5, IL-6, IL-17A, IL-10, IL-1, IL-1β, and IL-18-compared to those without DM (32, 106), contrasting with increased serum IL-1β in DM patients with severe TB (84). Most IL-20 subfamily cytokines (IL-19, IL-20, IL-22, IL-24) are significantly lower in TB-DM plasma, except IL-10, which is elevated (65), supported by reduced IL-22 in TB-DM patients (32, 57). Another study found raised IL-2, IFN-γ, and IL-17A in TB-DM without a corresponding drop in TGF-β; IL-10 remains increased in TB-DM versus TB alone (91). Notably, bronchoalveolar lavage fluid (BALF) from TB-DM patients shows higher immunosuppressive IL-10 but lower IFN-γ than TB patients without DM, suggesting differences in local lung versus systemic immune responses (73).

##### 3.1.2.2 Cytokine response to stimulation in TB-DM

Pro-inflammatory cytokines TNF, IFN-γ, IL-2, and IL-17A are elevated in TB-DM patients after whole blood antigen stimulation (31, 32, 68, 91). In PBMCs, *Mtb* stimulation increases IL-6 and IL-1β dose-dependently, while TNF rises only at highest glucose levels (92). MDMs from DM patients also secrete high IL-1β upon *Mtb* stimulation (76). Conversely, PBMCs stimulated with live *M. bovis* BCG show reduced IFN-γ and TNF linked to high glucose (83), and TB-DM patients exhibit decreased IL-1β, IL-6, and IFN-γ after *Mtb* stimulation (69), with sample type (MDMs vs. PBMCs) likely affecting IL-1β variability.

Other studies report reduced IFN-γ and IL-12 after *Mtb* antigen stimulation (94) and decreased IFN-γ from macrophages post-*Mtb* (59). Some find no significant changes in IFN-γ, TNF, IL-1Ra, IL-17, or IL-22 production after stimulation (69, 111). These conflicting findings may reflect differences in methodology, timing, medications, *Mtb* strains, disease severity, and metabolic or ethnic variations (114).

##### 3.1.2.3 Chemokines and chemokine receptors in TB-DM

Classical monocytes from TB-DM patients show increased CCR2 expression (109), potentially impairing lung trafficking. In another study, serum IP-10 levels were reduced in all TB+ groups, while SDF-1 remained elevated except in those with known DM and IL-8 levels decreased in newly diagnosed DM but increased in treated DM patients (104). These findings need to be validated, as the observed reduction in IP-10 in people with TB is in contrast to the majority of published data (119–121).

##### 3.1.2.4 Cytotoxic and immune markers in TB-DM

TB-DM individuals show reduced expression of cytotoxic markers (PFN, GZEB, CD107a) at baseline and after *Mtb* stimulation (68), and elevated high-sensitivity C- reactive protein (hs-CRP) in pleural effusion versus TB alone (115). High glucose conditions reduce HLA-DR and CD86 in MDMs (71). *Mtb*-stimulated MDMs from TB-DM patients also show lower CD64, CD206, and RAGE levels compared to TB-only, though RAGE does not differ significantly versus controls (62). While TLR-2 and TLR-4 expression on monocytes did not differ between DM patients and controls in whole blood, TLR expression increased under high glucose versus standard medium (80).

##### 3.1.2.5 Molecular markers and gene expression in TB-DM

The Molecular Distance to Health (MDH), a marker of immunological activation (122), is significantly higher in TB-DM patients than those without DM (81). TB-DM is also linked to elevated KEGG pathways involving immune-related proteins Integrin alpha M (ITGAM) and STAT1, which regulate cellular responses and leukocyte recruitment, indicating possible increased disease severity (107). TB patients, regardless of DM, show similar upregulation of type I and II interferon genes, but TB-DM differs markedly from DM-only patients (81). Conversely, another study found TB-DM patients had reduced IFN modules (especially type I interferon), along with downregulated NK cell and adaptive immune response modules (T cell activation, differentiation, B cell responses) compared to TB without DM (85). People with pre-DM or intermediate hyperglycaemia alongside TB also exhibit excessive gene expression exacerbation, similar to those with overt DM (85), indicating immunological changes occur early during the transition to DM.

##### 3.1.2.6 Innate and adaptive immune cell populations in TB-DM

TB-DM patients are more prone to lung cavities than those with TB alone, though managing fasting blood glucose may reduce this risk (79). They also exhibit higher neutrophil-to-lymphocyte (NLR) and monocyte-to-lymphocyte ratios (MLR) compared to TB-only patients (106). While CD4+ T, NK, and NKT cell counts are similar, TB-DM patients have reduced total T, CD8+ T, and B lymphocyte counts (79). BALF from TB-DM patients shows fewer CD4+CD25+ cells but more CD4+CD25+CD127- regulatory T cells (Tregs), linked to immune suppression and severe *Mtb* dissemination (73, 123). TB-DM patients also have lower frequencies of naive CD4+ T, naive and effector memory CD8+ T cells, myeloid and plasmacytoid DCs, and classical/intermediate monocytes, but increased central CD8+ T and classical memory B cells (78). *Mtb*-stimulated whole blood shows elevated monofunctional and dual-functional CD4+ Th1 and Th17 cells (63), though PBMCs from DM patients show a Th1 to Th2 shift after *Mtb* stimulation, indicating reduced Th1 immunity essential for intracellular bacterial defense (54).

##### 3.1.2.7 Phagocytosis and mycobacterial killing in TB-DM

Monocytes cultured in high glucose support increased *Mtb* growth vs. standard media, though intracellular *Mtb* growth remains similar between DM and non-DM subjects (80). In TB-DM patients, macrophage phagocytosis is impaired, with reduced vacuole formation and giant cell presence. High glucose further lowers *Mtb* adhesion and uptake, as seen in reduced CFU counts (59). Low vitamin D and glutathione levels also correlate with increased *Mtb* growth in monocytes and poor *Mtb* control in MDMs, respectively (108, 112). DM-induced phagocytic dysfunction is evident in macrophage-like promonocytic cells and MDMs, where elevated glucose correlates with reduced phagocytosis (110). *Mtb*-infected PBMCs exposed to high glucose also show diminished control of infection (63). While MDM binding to *Mtb* is slightly lower in DM patients, conditioned media from *Mtb*-infected DM MDMs yields CFU counts similar to non-DM control (76). Blood from DM patients stimulated with *Mtb* shows reduced phagocytosis by neutrophils and monocytes, though overall antimycobacterial activity is comparable to controls (75). Impaired *Mtb* co-localisation with LAMP-1/2 in THP-1 cells under high glucose further indicates defective phagocytosis and post-phagocytic killing (64).

##### 3.1.2.8 Endocrine and metabolic profiles in TB-DM

TB-DM is linked to altered adipokines-reduced adiponectin and adipsin, and elevated leptin-compared to TB without DM (72). DM-associated endocrine disruptions also affect TB immunity, with TB-DM patients showing higher cortisol and growth hormone levels (86, 87). Elevated resistin in DM is associated with reduced ROS production (PMA- and *M. marinum*-induced) and impaired inflammasome activation (84), aligning with increased oxidative stress markers (MDA, GSSG, ROS) in DM (112). Conversely, high glucose boosts ROS and NO production in *Mtb*-infected THP-1 cells (64), though *Mtb* may persist in macrophages even in the presence of NO (124).

##### 3.1.2.9 Immune and eicosanoid responses to treatment in TB-DM

Specific gene signatures may predict TB outcomes in TB-DM patients, offering early intervention markers (113). In TB-only patients, key immune genes (e.g., granulysin, GBP1) normalise within two weeks of anti-TB treatment (ATT), but responses are delayed in TB-DM (99). Interferon signalling, initially reduced in TB-DM, is later augmented during treatment (85). NK cell responses (elevated CD14, CD16) are higher pre-ATT in TB-DM; CD14 normalises, but CD16 remains elevated throughout treatment (97).

Before and 2 months into ATT, TB-DM patients show reduced classical/intermediate monocytes and plasmacytoid/myeloid DCs, which significantly increase by 6 months (101). T cells in TB-DM show lower naive/effector memory and higher central memory frequencies, with less change over time than in TB-only patients (78, 102). Reduced cell adherence may also contribute to worse outcomes in TB-DM (95).

IFN-γ and IL-12 take longer to normalise in TB-DM, while IL-15 remains suppressed at 2 months (94, 98). TB-DM patients show higher baseline inflammation and shifts in cytokines-lower pro-inflammatory cytokines, but elevated IL-17A, IL-17F, IL-5, IL-10, and TGF-β during/after ATT. Newly diagnosed DM patients have persistently lower IFN-γ, IL-2, and TNF than those with known DM (61, 100).

Eicosanoids (LXA4, 15-epi-LXA4, PGE2), linked to lung pathology and microbial burden, are elevated in TB-DM but decline with treatment (96, 125). GPR183, involved in mycobacterial control, is reduced in TB-DM but normalises after 6 months of ATT (116). Monocyte activation markers (sCD14, sCD163, STF) are elevated in TB-DM; sCD14 and STF remain high throughout treatment, while sCD163 declines after ATT initiation (77).

**Figure 2:**
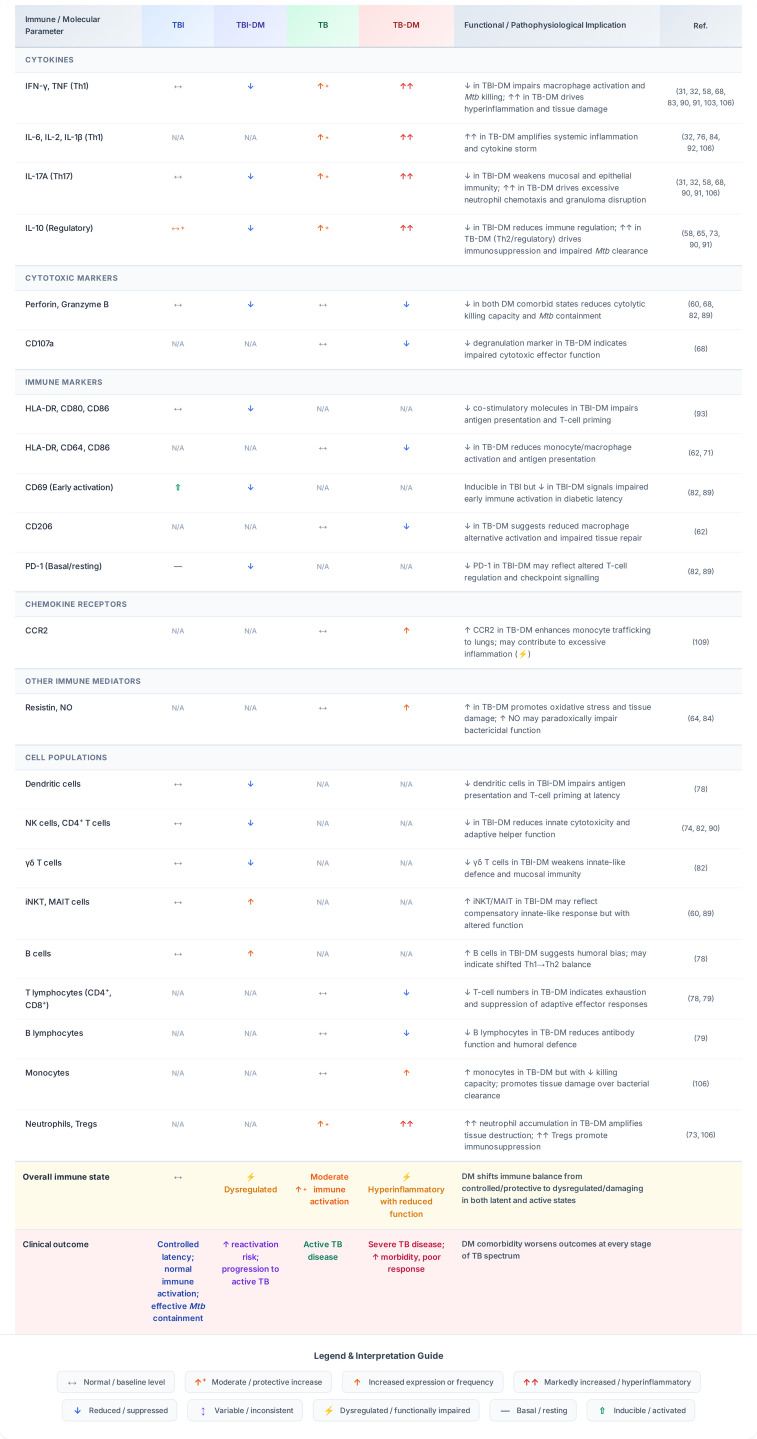
Comparison of immunity in individuals with TBI-only, TBI-DM, TB-only, and TB-DM. Individuals with TBI-DM predominantly exhibit underexpression of cytokines, cytotoxic, and immune markers, resulting in limited infiltration of inflammatory cells. Reduced expression of activation and antigen presentation markers contributes to a high bacterial load and impaired adaptive immune responses. Individuals with TB-DM predominantly exhibit overexpression of cytokines and underexpression of cytotoxic immune markers. Impaired phagocytosis and dysregulated inflammasome activation contribute to a high bacterial load. This is reflected in exaggerated immune cell infiltration and, subsequently, severe lung inflammation.

A schematic illustrating the immune response in individuals with TBI-only, TBI-DM, TB-only, and TB-DM is shown in **Figure 2**.

### 3.2 Animal models

#### 3.2.1 TBI and TB with coincident DM

**Supplementary Table 3** details the animal studies included in this review. Most TB-DM animal models use various mouse strains: BALB/c, C57BL/6, Ins2Akita, ICR, C3HeB/FeJ, and RAGE*−*/*−*. Each exhibits distinct immune responses to *Mtb* infection (126). For instance, C3HeB/FeJ best mimics human-like lesions and TB progression (127), while inbred strains like C57BL/6 and BALB/c generally tolerate *Mtb*, forming only proliferative, non-necrotic lesions (128–131) C57BL/6 mice also model latent TB, with slow disease progression and pulmonary damage emerging ~200 days post-infection (132), but rarely develop necrotic lesions seen in C3HeB/FeJ mice (133), -likely depending on *Mtb* strain (134). The Ins2Akita mouse, carrying a mutation in the insulin 2 gene, models T1DM through insulin deficiency and hyperglycaemia (135), enabling studies on how hyperglycaemia impacts TB susceptibility and immune response. ICR, a robust outbred strain, is widely used in infection and immune research. RAGE*−*/*−* mice, lacking the RAGE receptor, help explore its role in inflammation, immune modulation, and TB pathology. Still, mouse models often reflect chronic hyperglycaemia akin to total insulin deficiency in T1DM (51) limiting their relevance for T2DM, which comprises ~95% of DM cases (136). Moreover, they poorly model human granulomata and caseous necrosis. Guinea pigs, by contrast, more accurately mirror human TB’s clinical, immunological (51, 137, 138), and T2DM-related metabolic features (139).

##### 3.2.1.1 BALB/C mice

In a murine TBI-DM model (38), BALB/c mice infected with BCG and then *Mtb* via aerosol showed severe lung inflammation and lymphocytic infiltration, but no granulomas—unlike the poorly formed granulomas in TBI-only mice. TBI-DM mice also had higher lung bacterial loads. These immune disruptions correlated with elevated resuscitation-promoting factors (*Rpfs*) *B* and *D* (38), which support reactivation of dormant *Mtb* (140).

In the same model, diabetic TBI mice exhibited altered matrix metalloproteinase (MMP) expression: *mmp-*1 decreased early, then slightly increased, while *mmp-9* remained unchanged. Elevated MMP-9 in immunosuppressed settings may disrupt granuloma formation and promote *Mtb* reactivation in TBI (55). Additionally, BALB/c mice with DM injected intraperitoneally with *Mtb* showed significantly higher CD4 and CD8 levels than non-diabetic *Mtb*-infected mice, indicating more severe inflammation (40).

##### 3.2.1.2 C57BL/6 mice

Following low-dose aerosol infection with virulent *Mtb*, diabetic mice show reduced early macrophage and dendritic cell chemoattractants (47). Similarly, dysglycaemic mice on a high-fat diet (HFD) and infected with *Mtb* display impaired Cyp7b1-derived oxysterol production and disrupted GPR183 signalling, delaying early macrophage recruitment (52).

Diabetic mice infected intravenously with low/sub-lethal mycobacterial doses consistently exhibit dysregulated cytokines (reduced TNF, IL-6, MCP-1), higher bacillary loads, and more severe lesions (41–43). Likewise, chronic DM mice exposed to low-dose aerosol *Mtb* show heightened lung inflammation, with exaggerated leukocyte response and tenfold bacterial load increase versus controls (45). While early alveolar macrophage (AM) bacterial loads are similar between groups, moderate-dose aerosol infection leads to worsened progression and higher bacillary loads in DM mice over time (56).

Chronic DM also delays protective immunity, marked by fewer IFN-γ-producing lung T cells after low-dose *Mtb* challenge (45). This reflects late T cell priming in lymph nodes and lungs, resulting in lesions with highly infected AMs and fewer leukocytes, plus prolonged bacterial replication (47). Diabetes-associated reductions in CCL2 and CCL5 impair myeloid recruitment to infected macrophages (47). Additionally, hyperglycaemia disrupts AM sentinel function, limiting antigen-specific TNF+ CD4^+^ T cell activation after high-dose infection and adoptive transfer of AMs from DM to control mice (50).

Macrophage dysfunction, including reduced phagocytosis and killing, has been shown in BALF and peritoneal exudate-derived cells (41–43). Mortality increases with infection dose, reaching significance under lethal conditions (42, 43). Diabetic mice challenged with low-dose *Mtb* also show low IL-22 and high neutrophil elastase, contributing to epithelial damage and higher mortality (57). In another study using moderate-dose aerosol *Mtb*, all DM mice died while only 6.6% of controls did (56). This vulnerability was linked to IL-6-producing NK and CD11c^+^ cells; anti-IL-6 and anti-NK1.1 treatments improved survival and reduced lung pathology (56).

Interestingly, very low-dose *Mtb* infection reduces gut microbial diversity and increases lung bacterial loads in DM mice (30). Gut microbiota depletion impairs DC/AM function and Th1/Th17 responses, supporting *Mtb* persistence (141, 142). Moreover, mild-fat diet (MFD)-induced adiposity improves early containment-enhancing CD4^+^/CD8^+^ levels and reducing bacterial loads at day 30-and mitigates necrosis at day 60 via increased lipid degradation and oxidation (48).

##### 3.2.1.3 Ins2Akita and RAGE -/- mice

Low-dose aerosol *Mtb* infection in Ins2Akita mice showed a delayed but ultimately stronger IFN-γ response in chronic diabetic mice, with levels eventually exceeding those in euglycemic controls. However, *Mtb* growth in diabetic lungs remained poorly controlled. Inducible nitric oxide synthase expression was comparable between groups, indicating that diabetic lung macrophages still respond to IFN-γ and mount cell-mediated immunity (45).

While moderate-dose *Mtb* infection reduces CD14 and MARCO expression-impairing AM phagocytosis in wild-type C57BL/6 mice-chronic hyperglycaemia did not affect AM phagocytic function in RAGE-/- mice infected *in vitro*. This may reflect unchanged MARCO mRNA levels in BAL cells (50), suggesting a role for RAGE in shaping the diabetic AM phenotype.

##### 3.2.1.4 ICR and C3HeB/FeJ mice

Following high-dose intravenous *Mtb* infection in ICR mice, DM mice showed impaired T-cell function and reduced survival, with most dying within three months. In separate experiments, DM mice challenged intraperitoneally with moderate-to-high *P. aeruginosa* doses exhibited suppressed peritoneal macrophage phagocytosis, though macrophage killing after intravenous *Mtb* remained comparable to non-DM mice (46). These effects may stem from metabolic alterations and impaired antigen presentation.

In another model, high-dose BCG-vaccinated C3HeB/FeJ mice (which mimic human lesions) were fed HFD or ND and infected with low-dose aerosol *Mtb*. HFD mice showed gut dysbiosis, reduced bacteriome diversity, and a higher Firmicutes-to-Bacteroidetes ratio (44), associated with impaired immunity (143). This mirrors prior findings in C57BL/6 DM mice after very low-dose aerosol *Mtb* infection (30). HFD-fed mice had dampened responses to low/single infections but exaggerated inflammation (IFN-γ, TNF, IL-17, CXCL1, CXCL5) after high-dose or repeated exposures, suggesting HFD may accelerate TB progression (44).

##### 3.2.1.5 Rats

Low-dose aerosol *Mtb* infection in type 1 diabetic rats led to larger pulmonary granulomas than in non-diabetic controls. These lesions later fused and contained distinct foamy epithelioid macrophages, though lacked multinucleated giant cells or necrosis. Insulin treatment markedly reduced granuloma size. Diabetic rats also showed higher bacterial loads and elevated TNF, IFN-γ, and IL-1β mRNA expression, indicating heightened inflammation. Additionally, AMs from diabetic rats produced less NO, potentially impairing *Mtb* control (49).

##### 3.2.1.6 Guinea pigs

Low-dose aerosol *Mtb* exposure in Dunkin-Hartley guinea pigs led to increased pulmonary lesion burden, heightened bacilli shedding, AGE accumulation, elevated cytokines, and enhanced macrophage activation. This was accompanied by more CD45*high* CD4 T cells and greater neutrophil infiltration (51). Additionally, sucrose-fed guinea pigs showed higher serum FFAs and AGEs, and more severe lung lesions than water-fed controls (39).

**Figure 3:**
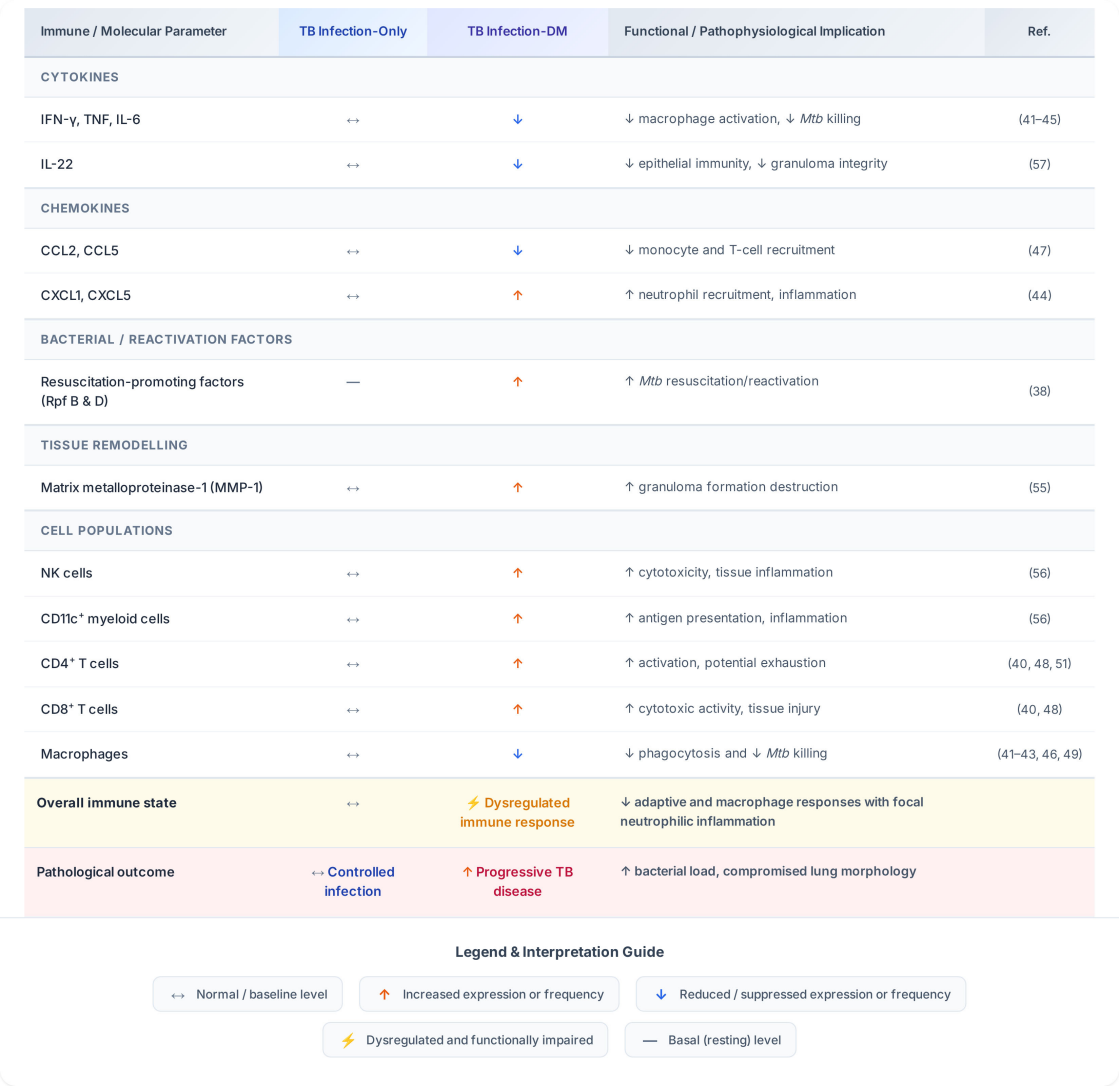
Comparison of immunity in animals with tuberculosis infection and concurrent DM. Diabetic animals with TB infection exhibit downregulated cytokine expression, reduced levels of CC chemokines, and elevated levels of CXC chemokines. The higher bacterial load contributes to severe inflammatory lesions and disruption of granuloma formation, which in turn accelerates the progression of TB disease.

A schematic illustrating the immune response in animals with TB infection and concurrent DM is shown in **Figure 3**.

## 4 Discussion

This review examines immune dysfunction in tuberculosis and DM comorbidity, incorporating human and animal studies. DM heightens TB susceptibility, worsens disease severity, delays recovery, and complicates treatment, with major implications for clinical management.

Human studies show that TBI-DM is associated with reduced cytokine, cytotoxic, and immune marker expression, along with lower frequencies of key innate and adaptive immune cells-potentially impairing cell-mediated immunity, phagosome maturation, and TB control compared to non-DM individuals. Conversely, TB-DM patients exhibit elevated cytokine levels and impaired inflammasome activation, contributing to increased bacterial burden, heightened immune cell infiltration, and severe lung inflammation.

Animal models reveal impaired immune responses marked by reduced cytokines, diminished CC chemokines, and compromised alveolar macrophage sentinel function. These defects likely hinder monocyte, macrophage, and T cell recruitment, while elevated CXC chemokines may drive excessive neutrophil infiltration. This highlights the increased susceptibility of diabetic hosts to mycobacterial infections, shaped by dose-dependent survival and dysregulated metabolic and immune systems. Data derived from animal models of TB-DM underpins translational research, and a recent study of human alveolar macrophages revealed epigenetic changes led to delayed immune responses, altered cytokine production and impaired neutrophil control (144).

Prior reviews have shown that metabolic dysfunction, including hyperglycaemia and dyslipidaemia, disrupts immune responses to *Mtb*. The 2017 review (145) highlighted oxidative stress, AGE–RAGE signalling, and potential targets like PPAR-γ and mTOR, noting metformin’s efficacy. A 2019 review (146) linked pre-DM and dyslipidaemia to impaired cytokine production and antigen presentation, with attention to vitamin D. A 2020 review (147) discussed how DM intensifies TB severity, inflammation, and resistance, and explored host-directed therapies, including IL-1β modulation.

Findings reinforce that TB-DM features hyperinflammation and poor *Mtb* control, while TBI-DM shows muted responses. These divergent immune profiles suggest stage-specific immune modulation by DM. A recent study also reported delayed *Mtb*-specific gene transcription and upregulated neutrophil-inhibitory genes in diabetic alveolar macrophages (144). Although some immune and eicosanoid imbalances may normalise during treatment, persistent alterations highlight the complex interplay between DM and TB outcomes. Even pre-DM or intermediate hyperglycaemia exacerbate TB pathology and impair immunity (85, 99, 148, 149). Transcriptomic studies have been at the forefront of the development of new biomarkers for TB diagnosis and TB treatment-response, yet the differences in gene expression profiles in TB patients with DM or pre-DM indicate care must be taken to include these populations in biomarker development (85, 122, 150, 151).

Despite shared mechanisms, several limitations of the available evidence should be considered. Study heterogeneity complicates interpretation, as variability in protocols, sample types, and the use of different mycobacterial stimuli (*Mtb* vs. BCG) influences findings. For instance, serum from coagulated blood shows elevated cytokines, while plasma better reflects *in vivo* states (152, 153). More rigorous and reproducible study design in TB-DM research is required to enable meta-analyses and to address the low or moderate quality of evidence in some studies. In addition, most experimental models in our review represent type 2-like DM (including STZ- and diet-induced models), insulin-deficient models such as Ins2Akita mice and the Komeda diabetes-prone rat reflecting type 1-like DM were less common, and the human studies focused on type 2 DM. Although we included a small number of type 1-like animal models that met our predefined eligibility criteria, these provide limited insight into shared TB-related immune alterations across different diabetic states and should be interpreted with caution. Finally, despite growing insights, significant research gaps remain. There is limited understanding of temporal immune changes during TB-DM progression, the relative contribution of hyperglycaemia versus dyslipidaemia, and how these metabolic perturbations interact with *Mtb*-specific immunity. Moreover, standardised methodologies and larger, multi-regional cohorts are required to validate and harmonise findings across diverse populations.

A new framework proposed by Dheda, Keertan et al. (154) categorises tuberculosis (TB) into five dimensions. This classification has the potential to reveal differences in immune responses across the TB spectrum. However, the studies reviewed did not classify TB cases according to this new framework. Future research applying this dimensional approach may provide deeper insights into the immune response at different stages of TB infection, particularly in the context of DM. Unlike TB-HIV, which is characterized by profound T-cell depletion, or TB-malnutrition, where nutrient deficiency drives immune exhaustion, TB-DM exhibits a paradoxical state of hyperinflammation alongside impaired pathogen control. Including comparator groups with only DM, without TB, would further clarify which immune changes are driven by DM itself.

This review underscores the importance of targeted metabolic and clinical management for people with TB-DM and suggest that this high-risk group may benefit from individualised clinical management and future evidence-based guidelines. Emerging observational and experimental data indicate that improved glycaemic control and lipid regulation may enhance immune function and treatment outcomes (48, 155), but studies directly linking metabolic optimisation to immune and clinical end-points in TB-DM remain limited. Host-directed therapies targeting inflammation and metabolism remain promising for future application, but currently available data are insufficient to support changes in routine clinical management. The synthesis of data presented here suggests that different and/or refined approaches may be required for host-directed therapy for TB in the diabetic host (reviewed in (156)). Thus, delineating TB-DM immune responses advances mechanistic understanding and may help inform the future design of precise immunometabolic interventions, as stronger interventional evidence becomes available.

In conclusion, this updated synthesis of TB-DM immunopathology refines our understanding of immune dysregulation in this comorbidity and identifies potential avenues for future therapeutic and biomarker research.

## Data Availability

The data analyzed in this study is subject to the following licenses/restrictions: The datasets can be shared with researchers upon request. Requests to access these datasets should be directed to Aminat Y. Saula, ays27@bath.ac.uk.
