# Supplementary material for: Immune dysregulation in tuberculosis-diabetes comorbidity: mechanistic and translational insights
Source: Front Immunol. 2026 Apr 23;17:1803046. doi: 10.3389/fimmu.2026.1803046 (PMC13149154; doi:10.3389/fimmu.2026.1803046)
Supplement: Supplementary file 1 [file Table1.docx]

**Table 1:** **Conditions for Studies on TBI-DM Comorbidity in This Systematic Review.** Abbreviations: pre-diabetes (pDM), healthy control (HC), protein-based assay (P), Flow cytometry (Flow cyt.), intracellular cytokine staining (ICS), enzyme-linked immunosorbent assay (ELISA), quantitative polymerase chain reaction (qPCR), cytometric bead assay (CBA), unstimulated (UNS), early secreted antigenic target of 6 kDa (ESAT-6), culture filtrate protein of 10 kDa (CFP-10), purified protein derivative (PPD), whole cell lysate (WCL), staphylococcal enterotoxin B (SEB), phorbol myristate acetate and ionomycin (P/I), not reported (NR), not applicable (NA), yes(Y), no (N), normal glucose tolerance (NGT), newly diagnosed diabetes mellitus (NWDM), and known diabetes mellitus (KDM).

| **Author (Year)** | **Age** | **Gender**  **(Male(M),**  **Female(F))** | **No of participants** | **Sample Collected, Methodology** | **Unstimulated, Stimulated Conditions** | **Controlled DM?** | **DM Medication** |
| --- | --- | --- | --- | --- | --- | --- | --- |
| Kumar et al. (2014) | TBI-DM (45.5 (28-68)), TBI (33.7 (19-60)), TBI-pDM (43.3 (23-65)), DM (45 (21-64)), HC (35.5 (20-54)) | TBI-DM (17 M), TBI (11 M), TBI-pDM (11 M), DM (14 M), HC (12 M) | 30 TBI-DM, 30 TBI, 30 TBI-pDM, 30 DM, 30 HC | Whole blood, P(ELISA, Bioplex) | UNS, *Mtb antigens* (ESAT-6, CFP-10, TB 7.7), Mitogen | NR | NA |
| Kumar et al. (2016) | TBI-DM (44 (26–65)), TBI (35 (19–63)) | TBI-DM (12 M, 10 F), TBI (11 M, 11 F) | 22 TBI-DM, 22 TBI | Whole blood, P(Flow cyt., ELISA, Bioplex) | UNS, *Mtb* antigens (PPD, ESAT6, CFP-10), Flu peptide pool | N | NA |
| Kumar et al. (2014) | TBI-DM (45.5 (28-68)), TBI (33.7 (19-60)), TBI-pDM (43.3 (23-65)), DM (45 (21-64)), HC (35.5 (20-54)) | TBI-DM (17 M), TBI (11 M), TBI-pDM (11 M), DM (14 M), HC (12 M) | 30 TBI-DM, 30 TBI, 30 TBI-pDM, 30 DM, 30 HC | Whole blood, P(ELISA, Bioplex) | UNS, *Mtb* antigens (ESAT-6, CFP-10, TB 7.7), Mitogen | NR | NA |
| Kumar et al. (2016) | TBI-DM (44 (26–65)), TBI (35 (19–63)) | TBI-DM (12 M, 10 F), TBI (11 M, 11 F) | 22 TBI-DM, 22 TBI | Whole blood, P(Flow cyt., ELISA, Bioplex) | UNS, *Mtb* antigens (PPD, ESAT6, CFP-10), Flu peptide pool | N | NA |
| Kumar et al. (2015) | TBI-DM (45 (28–65)), TBI (34 (19–60)) | TBI-DM (30 M, 14 F), TBI (25 M, 19 F) | 44 TBI-DM, 44 TBI | Whole blood, P(ELISA) | UNS | NR | NA |
| Aravindhan et al. (2022) | 30-60 | NR | 82 TBI-DM, 36 TBI, 95 DM, 43 HC | Whole blood, P(ELISA) | UNS | NR | NA |
| Bobhate et al. (2021) | NGT-TBI- (46.6±12.6), NGT-TBI+ (51.8±11.3), pDM-TBI- (50.3±10.1), pDM-TBI+ (49.8±9.1), NWDM-TBI- (48.9±12), NWDM-TBI+ (44.4±11.3), KDM-TBI- (54±9), KDM-TBI+ (52.8±9) | NGT-TBI- (16 M, 27 F), NGT-TBI+ (22 M, 14 F), pDM-TBI- (23 M, 14 F), pDM-TBI+ (16 M, 17 F), NWDM-TBI- (19 M, 12 F), NWDM-TBI+ (19 M, 6 F), KDM-TBI- (34 M, 13 F), KDM-TBI+ (36 M, 12 F) | 43 NGT-TBI-, 36 NGT-TBI+, 37 pDM-TBI-, 33 pDM-TBI+, 31 NWDM-TBI-, 25 NWDM-TBI+, 95 KDM-TBI-, 82 KDM-TBI+ | Whole blood, P(ELISA) | UNS | Y | NR |
| Aravindhan et al. (2022) | NGT-TBI- (45.3 ± 11.3), NGT-TBI+ (50. ± 10.9), pDM-TBI- (49.1 ± 10.2), pDM-TBI+ (49.9 ± 93), NWDM-TBI- (48.80 ± 11.3), NWDM-TBI+ (45.90 ± 10.7), KDM-TBI- (52.88 ± 95), KDM-TBI+ (53.14 ± 99) | NGT-TBI- (59 M, 57 F), NGT-TBI+ (33 M, 21 F), pDM-TBI- (91 M, 70 F), pDM-TBI+ (25 M, 23 F), NWDM-TBI- (82 M, 43 F), NWDM-TBI+ (30 M, 10 F), KDM-TBI- (120 M, 56 F), KDM-TBI+ (65 M, 19 F) | 116 NGT-TBI-, 54 NGT-TBI+, 161 pDM-TBI-, 48 pDM-TBI+, 125 NWDM-TBI-, 40 NWDM-TBI+, 176 KDM-TBI-, 84 KDM-TBI+ | Whole blood, P(ELISA), T(RNA, Gene exp, qPCR), Biochem.(TBARS, Griess) | UNS, *Mtb* antigens (*Mtb H3Rv*, PPD, WCL) | Y (only KDM) | NR |
| Kathamuthu et al. (2022) | TBI-DM (47.1 (25–60)), TBI (39.6 (24–62)), TBI-pDM (45.9 (25–62)) | TBI-DM (11 M, 9 F), TBI (10 M, 10 F), TBI-pDM (11 M, 9 F) | 20 TBI-DM, 20 TBI, 20 TBI-pDM | PBMCs, P(Flow cyt., ICS) | UNS, *Mtb* antigens (PPD, WCL), Mitogen(P/I) | NR | NA |
| Kathamuthu et al. (2022) | TBI-pDM (45.9 (25–62)), TBI-DM (47.1 (25–60)), TBI (39.6 (24–62)) | TBI-pDM (11 M, 9 F), TBI-DM (11 M, 9 F), TBI (10 M, 10 F) | 20 TBI-pDM, 20 TBI-DM, 20 TBI | PBMCs, P(Flow cyt., ICS) | UNS, *Mtb* antigens (PPD, WCL), P/I | NR | NA |
| Kathamuthu et al. (2021) | TBI-DM(47.1 (25-60)), TBI (39.6 (24-62)), TBI-pDM (45.9 (25-62)) | TBI-DM (11 M, 9 F), TBI (10 M, 10 F), TBI-pDM (11 M, 9 F) | 20 TBI-DM, 20 TBI, 20 TBI-pDM | PBMCs, P(Flow cyt., ICS, ELISA) | UNS, *Mtb* antigens (PPD, WCL), P/I | NR | NA |
| Ssekammate et al. (2021) | TBI-DM (51 (40-55.5)), 10 TBI (42 (38-49)), 14 DM (55 (41.5-57)), 11 HC (34 (22-40)) | TBI-DM (5 M, 8 F), 10 TBI (4 M, 10 F), 14 DM (5 M, 5 F), 11 HC (3 M, 8 F) | 13 TBI-DM, 10 TBI, 14 DM, 11 HC | PBMCs, P(Flow cyt.) | P/I | N | NA |
| Magee et al. (2020) | > 19 years | NR | 513 TBI-DM | 2011-2012 NHANES data, P(QFT) | UNS | NR | NR |
| Kumar et al. (2016) | TBI-DM (44 (26–65)), TBI (35 (19–63)) | TBI-DM (12 M, 10 F), TBI (11 M, 11 F) | 22 TBI-DM, 22 TBI | Whole blood, P(Flow cyt., ICS) | *Mtb* antigens (PPD, ESAT-6, CFP-10 ), P/I | N | NA |
| Kumar et al. (2015) | TBI-DM (47 (33–72)), TBI (33 (19–60)), DM (47 (33–72)), HC (33 (19–60)) | TBI-DM (27 M, 23 F), TBI (31 M, 19 F), DM (28 M, 22 F), HC (21 M, 29 F) | 50 TBI, 50 DM, 50 HC | Whole blood, P(Flow cyt.) | NA (except monoclonal antibodies) | NR | NA |
| Kumar et al. (2016) | TBI-DM (45 (28–65)), TBI (44 (29–60)) | TBI-DM (30 M, 14 F), TBI (29 M, 15 F) | 44 TBI-DM, 44 TBI | Plasma, P(Bioplex) | UNS | N | NA |
| Boillat-Blanco et al. (2018) | TBI-DM (45 (27)), TBI (35 (10)) | TBI-DM (5 Males), TBI (10 Males) | 12 TBI-DM, 14 TBI | PBMCs, P(Flow cyt., ICS, ELISPOT) | *M. bovis* BCG, *Mtb* antigens (ESAT6, CFP-10), SEB | Y | Glibenclamide, + Metformin, Insulin |
| Lopez-Lopez et al. (2018) | DM (56 (8.97)), HC (44.78 (5.47)) | DM (2 M), HC (1 M) | 10 DM, 9 HC | Whole blood & PBMCs, P(Flow cyt., CBA, Ziehl-Neelson) | UNS, *Mtb* (*H37Rv*, EA phenotype 1, Haarlem phenotype 2, Haarlem phenotype 4) | Y | Glibenclamide, Metformin |
