# Supplementary material for: Immune dysregulation in tuberculosis-diabetes comorbidity: mechanistic and translational insights
Source: Front Immunol. 2026 Apr 23;17:1803046. doi: 10.3389/fimmu.2026.1803046 (PMC13149154; doi:10.3389/fimmu.2026.1803046)
Supplement: Supplementary file 2 [file Table2.docx]

**Table 2:**  **Conditions for Studies on TB-DM Comorbidity in This Systematic Review.** Abbreviations: pre-diabetes (pDM), healthy control (HC), household contacts (HHC), protein-based assay (P), transcriptome assay (T), functional assay (F), Flow cytometry (Flow cyt.), intracellular cytokine staining (ICS), enzyme-linked immunosorbent assay (ELISA), quantitative polymerase chain reaction (qPCR), cytometric bead assay (CBA), gene ontology (GO), dual-colour reverse-transcriptase multiplex ligation-dependent probe amplification (dcRT-MLPA), unstimulated (UNS), early secreted antigenic target of 6 kDa (ESAT-6), culture filtrate protein of 10 kDa (CFP-10), purified protein derivative (PPD), whole cell lysate (WCL), phorbol 12-myristate 13-acetate (PMA), staphylococcal enterotoxin B (SEB), phorbol myristate acetate and ionomycin (P/I), not reported (NR), not applicable (NA), yes(Y), no (N), normal glucose tolerance (NGT), newly diagnosed diabetes mellitus (NWDM), and known diabetes mellitus (KDM), length of hospital stay (LOS), Revised National Tuberculosis Control Programme (RNTCP), anti-tuberculosis treatment (ATT), directly observed treatment, short-course (DOTS), interferon-gamma release assay (IGRA), chemiluminescent immunoassay (CICA), enzyme-linked immunospot assay (ELISPOT), reactive oxygen species (ROS), lactate dehydrogenase (LDH), bicinchoninic acid assay (BCA), quantitative reverse transcription polymerase chain reaction (qRT-PCR), concanavalin A (Con-A), lipopolysaccharide (LPS), 3-(4,5-dimethylthiazol-2-yl)-2,5-diphenyltetrazolium bromide assay (MTT).

| **Author (Year)** | **Age** | **Gender**  **(Male(M),**  **Female(F))** | **No of participants** | **Sample Collected, Methodology** | **Unstimulated, Stimulated Conditions** | **Controlled DM?** | **DM Med.** | **Controlled TB?** | **ATT Regimen** | **ATT Comparison** |
| --- | --- | --- | --- | --- | --- | --- | --- | --- | --- | --- |
| Antari et al. (2018) | NR | NR | 30 TB-DM, 30 TB | PBMCs, F(CFU) | *Mtb H37Rv* ATCC 27294ᵀ | NR | NA | NR | NA | N |
| Kumar et al. (2013) | TB-DM (45 (33–70)), TB (43.5 (20–70)) | TB-DM (31 M, 13 F), TB (38 M, 6 F) | 44 TB-DM, 44 TB | Plasma, Whole blood, P(ELISA, Multiplex) | UNS, *Mtb* antigens (ESAT6, CFP-10, TB 7.7), P/I | NR | NA | N | NA | N |
| Kumar et al. (2020) | TB-NWDM (42 (29–70)), TB-KDM (52 (25–70)), TB (41 (24–67)), DM (44(33–59)) | TB-NWDM (18 M, 12 F), TB-KDM (20 M, 10 F), TB (17 M, 13 F), DM (20 M, 10 F) | 30 TB-NWDM, 30 TB-KDM, 30 TB, 30 DM | Whole blood, P(Bioplex, Multiplex) | UNS, *Mtb* antigens (ESAT6, CFP-10, TB 7.7), P/I | NR | NA | Y | Standard ATT | Y (0, 2, 6 months) |
| Arce-Mendoza et al. (2008) | ≥ 20 years | 21 M, 39 F | 15 TB-DM, 15 TB, 15 DM, 15 HC | Blood, PBMCs, MDMs, P(Flow cyt.) | UNS, *Mtb H37Rv* | Y | NR | Y (3 months) | NA | N |
| Bobadilla-del-Valle et al. (2021) | Opt. GC (56.2 ± 11.75), Poor GC (52.05 ± 9.94), HC (42.12 ± 11.75) | NR | 54 Opt. GC, 35 Poor GC, 44 HC | Blood, F(MGIT), P(Flow cyt.) | UNS, *Mtb H37Rv* | N | NR | N | NA | N |
| Kundu et al. (2021) | NR | NR | NR | THP-1 cell, F(Griess, MTT), P(Flow cyt, C-M) | *Mtb H37Rv* | NR | NA | N | NA | N |
| Kumar et al. (2015) | TB-DM (49(40–58)), TB (45(40–55)) | TB-DM (31 M, 13 F), TB (38 M, 6 F) | 44 TB-DM, 44 TB | Whole blood, P(ELISA) | UNS | NR | NA | N | NA | N |
| Kumar et al. (2015) | TB-DM (49.1(40–58)), TB (45.4(40–55)) | TB-DM (16 M, 6 F), TB (18 M, 5 F) | 22 TB-DM, 22 TB | Whole blood, P(Flow cyt.) | UNS | NR | NA | N | NA | N |
| Lachmandas et al. (2018) | DM (48.1 ±2.7), HC (47.5±2.6) | NR | 24 DM, 24 HC | PBMCs, T(qPCR, RNA), P(ELISA) | *Mtb* antigens (ESAT-6, CFP-10), anti-CD3 | Y | Insulin (≥1 year) | N | NA | N |
| Monroy-Merida et al. (2021) | NR | NR | NR | PBMCs, F(WST-1), P(Flow cyt, C-M) | *Mtb H37Ra*, LPS | Y (using 11 mM vs 30 mM glucose conc.) | NR | NR | NA | N |
| Kumar et al. (2016) | TB-DM (49(40–58)), TB (45(40–55)) | TB-DM (31 M, 13 F), TB (30 M, 14 F) | 44 TB-DM, 44 TB | Plasma, P(Bioplex) | UNS | NR | NA | N | NA | N |
| Sun et al. (2012) | TB-DM (52(19)), TB-pleurisy(45 (18)), TB (45(15)), HC (43(14)) | TB-DM (26 M), TB-pleurisy (22 M), TB (25 M), HC (20 M) | 30 TB-DM, 30 TB-pleurisy, 30 TB, 30 HC | BALF, Blood, Pleural effusion, P(ELISA, Flow cyt.) | UNS | NR | NA | NR | NA | N |
| Restrepo et al. (2008) | ≥20 years | 42 M, 24 F | 29 TB-DM, 37 TB | Whole blood, P(ELISA, Multiplex) | PPD from *Mtb*, superantigen control | Y (poor control using HbA1c level) | NR | Y (ATT ≤8 days) | NR | N |
| Raposo-García et al. (2017) | DM (73.1 SD 11.2), nonDM(68.4 SD 16.2) | DM (24 M, 17 F), nonDM (19 M, 21 F) | 41 DM, 40nonDM | Whole blood, P(ELISA, Flow cyt.), CFU | UNS, *Mtb HL186T* | Y (poor adherence) | NR | N | NA | N |
| Valtierra-Alvarado et al. (2021) | DM (47.79±7.61), HC (42.39±7.38) | NR | 14 DM, 18 HC | Monocytes (from Buffy coat), P(FCAP, Flow cyt.), CFU | *Mtb H37Rv* | Y | Metformin, Glibenclamide | N | NA | N |
| Kumar et al. (2019) | TB-KDM (52(25–70)), TB-NWDM (42 (29–70)), TB (39 (24–67)), DM (44 (33–68)), HC (34 (23–55)) | TB-KDM (16 M, 6 F), TB-NWDM (18 M, 4 F), TB (27 M, 17 F), DM (30 M, 14 F), HC (15 M, 15 F) | 22 TB-KDM, 22 TB-NWDM, 44 TB, 44 DM, 30 HC | Plasma, P(ELISA) | UNS | Y (in TB-KDM) | Metformin | Y | Standard ATT using DOTS | Y (0, 2, 6 months) |
| Kumar et al. (2015) | TB-DM (44(33–70)), TB (45(28–65)), DM (47(33–72)), HC (33(19–60)) | TB-DM (31 M, 19 F), TB (27 M, 23 F), DM (28 M, 22 F), HC (21 M, 29 F) | 50 TB-DM, 50 TB, 50 DM, 50 HC | Whole blood, P(Flow cyt.) | NA (except monoclonal antibodies) | NR | NA | N | NA | N |
| Wei et al. (2022) | TB-DM (59.68±14.33), TB (44.80±20.00) | TB-DM (127 M, 41 F), TB (113 M, 46 F) | 168 TB-DM, 159 TB | Blood, P(IGRA, ELISA, Flow cyt., CICA) | NA | NR | NA | Y | Standard ATT | Y (LOS > 2 wks, LOS≤2 wks) |
| Torres et al. (2019) | DM (53 [30-68]), HC (48 [33-65]) | DM (15 M, 28 F), HC (9 M, 17 F) | 43 DM, 26 HC | Whole blood, P(Flow cyt., TLR ligands), CFU | *Mtb H37Ra*, Pam3Cys,LPS | NR | NA | NR | NA | N |
| Prada-Medina et al. (2017) | TB-DM (49 [39-54]), TB (39 [31-52]), DM (52 [45-61]), HC (40 [31-48]) | TB-DM (20 M), TB (27 M), DM (22 M), HC (24 M) | 30 TB-DM, 30 TB, 30 DM, 30 HC | Plasma, whole blood, T(RNAseq, Gene exp.) | NA | Y | NR | Y (< 7 day) | NR | N |
| Al-Attiyah et al. (2009) | TB-DM (20–57), TB (28–57), HC (20–47) | TB-DM (10 M, 1 F), TB (17 M, 1 F), HC (18 M, 2 F) | 11 TB-DM, 18 TB, 20 HC | Venous blood, P(Flow cyt.) | UNS, *M. bovis* BCG, *Mtb* antigens (*Mtb* H37Rv, MT-CF, MT-CW), Peptide pools (RD1, RD4, RD6, RD10) | N | NA | NR | NA | N |
| Boillat-Blanco et al. (2018) | TB-DM (57 [7]), TB (28 [11]) | TB-DM (7 M), TB (17 M) | 8 TB-DM, 20 TB | PBMCs, P(Flow cyt., ICS, ELISPOT) | *M. bovis* BCG, *Mtb* antigens (ESAT6, CFP-10), SEB | Y | Glibenclamide, Metformin, Insulin | N | NA | N |
| Chao et al. (2015) | Mild TB (54±23), Severe TB (59±19), DM (61±11), HC (58±13) | Mild TB (58 M), Severe TB (36 M), DM (47 M), HC (42 M) | 102 Mild TB (13 TB-DM), 49 Severe TB (20 TB-DM), 71 DM, 75 HC | Venous blood, P(ELISA), ROS detection | UNS, *M. marinum* | NR | NA | NR | NA | N |
| Fernández et al. (2020) | TB-DM (50 (41–62)), TB (41 (34–59)), DM (54 (51–61)), HC (45 (35–62)) | TB-DM (5 M, 6 F), TB (13 M, 8 F), DM (8 M, 10 F), HC (13 M, 9 F) | 11 TB-DM, 21 TB, 18 DM, 22 HC | Whole blood, T(qPCR, RNA, CDA), P(ELISA, CBA) | UNS, *Mtb H37Rv* | NR | NA | N | NA | N |
| Fernández et al. (2016) | TB-DM (53(46–62.7)), TB (52 (30–62)), HC (47 (39.5–51.7)) | TB-DM (11 M, 3 F), TB (18 M, 3 F), HC (17 M, 3 F) | 14 TB-DM, 21 TB, 20 HC | Blood, P(ELISA, Proliferation assay) | UNS, *Mtb H37Rv* | NR | NA | N | NA | N |
| Gomez et al. (2013) | DM (27–61), nonDM (25–56) | NR | NR | Blood, Host-pathogen | *Mtb H37Rv* | N | NA | NR | NA | N |
| Kumar et al. (2013) | TB-DM (49.1(40–58)), TB (45.4(40–55)) | TB-DM (16 M, 6 F), TB (18 M, 5 F) | 22 TB-DM, 22 TB | Whole blood, P(ELISA, Flow cyt.), F | UNS, *Mtb* antigens (PPD, ESAT6, CFP-10), anti-CD3 | NR | NA | N | NA | N |
| Lachmandas et al. (2015) | NR | NR | NR | PBMCs (from buffy coats), P(ELISA, BCA), F(LDH, Phagocytosis assay) | *Mtb H37Rv* lysate, heat-killed Candida albicans, LPS | NR | NA | NR | NA | N |
| Eckold et al. (2021) | South Africa: TB-DM (46 (27–57)), TB (48 (31–56)), DM (49 (29–64)), TB-IH (44.5 (25–57)), HC (42 (30–70)); Indonesia: TB-DM (52 (33–66)), TB (47 (28–62)), TB-IH (51 (37–54)); Peru: TB-DM (50.5 (42–58)), TB (55 (31–69)), TB-IH (52 (31–68)); Romania: TB-DM (47 (22–64)), TB (43 (30–64)), DM (55 (38–65)), TB-IH (48.5 (22–63)), HC (46 (38–61)) | South Africa: TB-DM (7 M, 8 F), TB (2 M, 9 F), DM (15 M, 18 F), TB-IH (12 M, 8 F), HC (12 M, 12 F); Indonesia: TB-DM (11 M, 8 F), TB (7 M, 7 F), TB-IH (4 M, 1 F); Peru: TB-DM (6 M, 6 F), TB (5 M, 6 F), TB-IH (5 M, 4 F); Romania: TB-DM (13 M, 2 F), TB (6 M, 4 F), DM (14 M, 5 F), TB-IH (9 M, 1 F), HC (10 M, 2 F) | South Africa: 15 TB-DM, 11 TB, 33 DM, 20 TB-IH, 24 HC; Indonesia: 19 TB-DM, 14 TB, 5 TB-IH; Peru: 12 TB-DM, 11 TB, 9 TB-IH; Romania: 15 TB-DM, 10 TB, 19 DM, 10 TB-IH, 12 HC | Whole blood, T(RNASeq) | NR | NR | NA | N | NA | N |
| Meenakshi et al. (2016) | NR | NR | 50 TB-DM, 50 TB-DM-HHC, 50 TB, 50 TB-HHC, 50 DM, 50 HC | Blood, T(RT-PCR gene exp.), P(ELISA), F(MTT) | *Mtb* H37rvAg 85A antigen, Con-A | NR | NA | Y | RNTCP | Y (0, 4, 6, 12, months) |
| Mendoza et al. (2012) | TB-DM (56.7 (34-69)), TB (48.7 (24-66)), DM (56.07 (30-81)), HC (33.48 (19-51)) | TB-DM (5 M, 5 F), TB (5 M, 5 F), DM (10 M, 20 F), HC (27 M, 17 F) | 10 TB-DM, 10 TB, 30 DM, 44 HC | Peripheral blood, F | PMA | Y | Glibenclamide, Metformin | Y | NR | N |
| Kumar et al. (2019) | TB-DM (47 (25-70)), TB (39 (24-67)), DM (44 (33-68)), HC (34 (23-55)) | TB-DM (34 M, 10 F), TB (27 M, 17 F), DM (30 M, 14 F), HC (15 M, 15 F) | 44 TB-DM, 44 TB, 44 DM, 30 HC | Plasma, P(ELISA) | UNS | NR | NA | Y (after recruitment) | Standard ATT (DOTS) | Y (0, 6 months) |
| Ponnana et al. (2020) | TB-DM (42.4±13.4), TB (34.0±13.80), DM (46.5±15.10), HC (25.0±8.9), HHC (34.0±13.01) | TB-DM (19 M, 6 F), TB (16 M, 9 F), DM (13 M, 12 F), HC (12 M, 13 F), HHC (10 M, 15 F) | 25 TB-DM, 25 TB, 25 DM, 25 HC, 25 HHC | Venous blood, P(Flow cyt.) | NR | NR | NA | Y | RNTCP | Y (0, 6, 12 months) |
| Sánchez-Jiménez et al. (2018) | TB-DM (48.5±13.7), TB (51.0±14.2), HC-DM (60.5±9.7), HHC (46.3±15.1) | TB-DM (17 M, 8 F), TB (5 M, 20 F), HC-DM (8 M, 17 F), HHC (15 M, 31 F) | 25 TB-DM, 25 TB, 25 HC-DM, 46 HHC | Blood, P(ELISA), F | NR | Y | Glibenclamide, Metformin, Insulin | Y (≥ 2 months) | RIF, INH, PZA, EMB | N |
| Eckold et al. (2023) | NR | NR | Indonesia: 44 TB-DM, 20 TB, 0 TB-preDM, 19 TB-rel-IH; South Africa: 19 TB-DM, 17 TB, 28 TB-preDM, 32 TB-rel-IH, 27 HC; Romania: 5 TB-DM, 5 TB, 5 TB-preDM, 2 TB-rel-IH | Venous blood, T(RNASeq, Gene exp.) | NR | Y | Local standard care of treatment | Y | Standard first-line TB treatment | 2, 8 wks, 6, 12, 18 months |
| Kumar et al. (2019) | India: TB-DM (46 (38–52)), TB (39.5 (30–47)) Brazil: TB-DM (45 (30.5–49.5)), TB (46 (37–56)) | India: TB-DM (32 M), TB (37 M) Brazil: TB-DM (13 M), TB (13 M) | India: 43 TB-DM, 44 TB Brazil: 26 TB-DM, 25 TB | Plasma | Plasma mediators, P(ELISA, Multiplex) | NR | NA | Y | India: Drug-sensitive treatment Brazil: standard treatment | India: Monthly (0-6 months), Quarterly (up to 18 months) Brazil: 0, 2, 6 months |
| Kumar et al. (2016) | TB-DM (48 (25–70)), TB (40 (25–67)) | TB-DM (23 M, 7 F), TB (24 M, 3 F) | 30 TB-DM, 27 TB | Blood, P(Flow cyt.), F | Monoclonal antibodies | NR | NA | Y | RIPE for 2 months; INH, RIF for 4 months | Y (0, 2, 6 months) |
| Kumar et al. (2016) | TB-DM (48 (25–70)), TB (40 (25–67)) | TB-DM (23 M, 7 F), TB (24 M, 3 F) | 30 TB-DM, 27 TB | Whole blood, P(Flow cyt.), F | Monoclonal antibodies | NR | NA | Y | RIPE for 2 months; INH, RIF for 4 months | Y (0, 2, 6 months) |
| Aravindhan et al. (2018) | NGT-LTB- (42±9), NGT-LTB+ (37±14), pDM-LTB- (45±10), pDM-LTB+ (40±14), NWDM-LTB- (46±9), NWDM-LTB+ (51±12), KDM-LTB- (51±9), KDM-LTB+ (51±11) | NGT-LTB-(105 M, 95 F), NGT-LTB+(75 M,33 F), pDM-LTB-(108 M,77 F), pDM-LTB+(71 M,34 F), NWDM-LTB-(69 M,36 F), NWDM-LTB+(48 M,15 F), KDM-LTB-(77 M,54 F), KDM-LTB+(82 M,26 F) | 200 NGT-TB-, 108 NGT-TB+, 185 pDM-TB-, 105 pDM-TB+, 105 NWDM-TB-, 63 NWDM-TB+, 131 KDM-TB-, 108 KDM-TB+ | Venous blood, P(ELISA), P-based functional assay (lipids) | NR | Y (only KDM) | NA | Y | DOTS | N |
| Chen et al. (2022) | TB-DM (60.15 (12.25)), TB (58.15 (12.00)), DM (59.91 (10.50)), HC (59.90 (8.25)) | TB-DM (8 M), TB (9 M), DM (8 M), HC (8 M) | 12 TB-DM, 14 TB, 12 DM, 12 HC | Peripheral blood, P(ELISA), F(WBC count) | NR | NR | NA | NR | NA | N |
| Xue et al. (2019) | TB-DM (18-76), TB (19-75) | TB-DM (38 M, 22 F), TB (40 M, 20 F) | 60 TB-DM, 60 TB | Pleural effusion, P(ELISA, immuno-turbidimetry) | TNF, hs-CRP | NR | NA | NR | NA | N |
| Cheekatla et al. (2016) | NR | NR | 20 TB-DM, 20 TB, 20 HC | Blood, P(ELISA, Flow cyt.), T(qPCR) | UNS, PPD, anti-IL-6 antibody | NR | NR | Y (< 1 wk) | NR | N |
| Tripathi et al. (2019) | TB-DM (30-55), TB (18-43) | TB-DM (6 M, 8 F), TB (12 M, 20 F) | 14 TB-DM, 32 TB | Blood, P | UNS | NR | NR | N | NA | N |
| Verma et al. (2023) | NR | NR | NR | THP-1 cell cult., T(qRT-PCR), P(CBA), F(mac. infection) | *Mtb H37Rv* | NR | NA | NR | NA | N |
| Selvan et al. (2022) | NR | NR | NR | Blood/serum gene expr. (PMID:28,515,464), T(RNA Seq), F(GO, KEGG), P | NR | NR | NA | NR | NA | N |
| Herrera et al. (2017) | DM (49.4±7.7), HC (44.8±8.0) | DM (17 M, 34 F), HC (12 M, 26 F) | 51 DM, 38 HC | Blood, T(qPCR), F(CFU), P(Vit. D) | *Mtb* H37Ra | NR | NA | NR | NA | N |
| Stew et al. (2013) | NR | 19 M, 13 F | 16 TB-DM, 16 TB | PBMCs, P(Flow cyt.) | NR | NR | NA | Y (≤3 days) | NR | N |
| Montoya-Rosales et al. (2016) | NR | NR | NR | Human U937 promonocytic cell line, T(RNA Seq, PCR), F | *Mtb H37Rv*, PMA, glucose conc. (11 mM, 16 mM, 22 mM) | NR | NA | NR | NA | N |
| Stalenhoef et al. (2008) | TB-DM (47.4±7.2), TB (43.0±7.1), DM (49.1±7.0), HC (45.0±8.6) | TB-DM (13 M), TB (21 M), DM (13 M), HC (20 M) | 23 TB-DM, 34 TB, 32 DM, 36 HC | Blood, P(ELISA, Bioplex) | *Mtb* sonicate, LPS, PHA | NR | NA | N | NA | N |
| Lagman et al. (2015) | 25-65 yrs | NR | 10 DM, 10 HC | Whole blood, P(ELISA, GSH), F(ROS, mac. infection) | *Mtb H37Rv*, GSH-enhancing agents (NAC, lGSH) | N | NA | N | NA | N |
| Van et al. (2022) | South Africa: Combined TB groups (45 (19-68)), HC (42 (30-70)). Indonesia: Combined TB groups (49 (25-73)). Validation cohort (India): 43 (18-75). | South Africa: 56 M, 38 F. Indonesia: 45 M, 36 F. Validation cohort (India): 55 M, 12 F. | South Africa: 17 TB-DM, 60 TB-AH, 17 TB Indonesia: 42 TB-DM, 19 TB-IH, 20 TB. Validation cohort (India): 67. | Blood, T(dCRT-MLPA, RNASeq) | RNA-Seq, dcRT-MLPA | Y | Metformin, Glibenclamide | Y | Standard first-line regimen | Y (0,2 wks, 2, 12 months) |
| Bartlett et al. (2020) | NR | NR | 9 TB, 7 TB-DM | Blood, PBMCs, T(RNA, Nano string), F(CFU), P(western blotting, immunofluorescence) | *Mtb H37Rv*, *M. bovis* BCG, 7α,25-dihydroxycholesterol, and/or GSK682753 | NR | NA | Y | Standard TB treatment | Y (0,6 months) |
| Restrepo et al. (2018) | 30-65 yrs | Texas-Mexico: 35 M, 95 F. South Africa: 28 M, 67 F | Texas-Mexico: 32 TB-DM, 72 TB South Africa: 41 TB-DM, 54 TB | Blood, F | NR | NR | NA | N | NA | N |
