# Supplementary material for: Immune dysregulation in tuberculosis-diabetes comorbidity: mechanistic and translational insights
Source: Front Immunol. 2026 Apr 23;17:1803046. doi: 10.3389/fimmu.2026.1803046 (PMC13149154; doi:10.3389/fimmu.2026.1803046)
Supplement: Supplementary file 3 [file Table3.docx]

**Table 3:** **Characteristics of the Animal Models in this Systematic Review.** Abbreviations: Not reported (NR), resident peritoneal cells (RPC), bronchoalveolar lavage (BAL) cells, bronchoalveolar lavage fluid (BALF), streptozotocin (STZ), nicotinamide (NA), colony-forming units (CFU), enzyme-linked immunosorbent assay (ELISA), quantitative polymerase chain reaction (qPCR), high-fat diet (HFD), energy-dense diet (EDD), medium-fat diet (MFD), intraperitoneal (i.p.), intravenous (i.v.), subcutaneous (s.c.), lymph nodes (LNs), protein-based assay (P), transcriptome analysis (T), functional assay (F), Flow cytometry (Flow cyt.), intracellular cytokine staining (ICS), cytometric bead assay (CBA), and histopathology (H.path.), enzyme-linked immunospot assay (ELISPOT), reverse transcription polymerase chain reaction (RT-PCR), quantitative reverse transcription polymerase chain reaction (qRT-PCR).

| **Author (Year)** | **Animal (strain)** | **Age** | **Gender**  **(Male(M),**  **Female(F))** | **No of Animals** | **DM Induction** | **Antigens & Stimulation** | **Infection Dose** | **Infection Route** | **Sample Collected, Methodology** |
| --- | --- | --- | --- | --- | --- | --- | --- | --- | --- |
| Verma et al. (2021) | Mice (BALB/c) | 5-6 wks | NR | 72 | STZ | BCG, *Mtb H37Rv* | O.D. 1.1 at 600nm ~3.1 × 10^7 | Aerosol | Lungs, Spleen, T(RNA, qRT-PCR), F. (CFU, H-Path.) |
| Verma et al. (2023) | Mice (BALB/c) | 5-6 wks | NR | 72 | STZ | BCG, *Mtb H37Rv* | O.D. 1.1 at 600nm | Aerosol | Lungs, Spleen, Serum, T(qRT-PCR) P(CBA, ELISA), F (H & E, CFU) |
| Agustin et al. (2021) | Mice (BALB/c) | 6-8 wks | F | 30 | i.p STZ | *Mtb*, MDR-TB | 0.2 mL Mtb | i.p injection | Serum, PBMC, F |
| Vallerskog et al. (2010) | Mice (C57BL/6) | ≥ 8 wks | NR | NR | i.p. STZ | *Mtb* Erdman, Ag85 peptide | ~100 CFU | Aerosol | Lungs, LNs, F P(Flow cyt., ELISA, ELISPOT) |
| Ngo et al. (2022) | Mice (C57BL/6, GPR183KO) | 6 wks | M | NR | HFD | *Mtb H37Rv* | ~150 CFU | Aerosol | Lungs, Blood, T(qRT-PCR), P(IHC), F(metabolite assay) |
| Alim et al. (2017) | Mice (C57BL/6) | 6 wks | M | NR | EDD | *M. fortuitum,* Mycolic acid | 1 × 10^7 CFU | i.v. injection | Lungs, Spleen, Liver, RPC, BAL cells, P(CBA, Flow cyt.), F (Phagocytosis, killing assay) |
| Alim et al. (2019) | Mice (C57BL/6) | 6 wks | M | NR | EDD | *M. bovis* BCG | 1 × 10^6 CFU, 2 × 10^6 CFU | i.v.injection | Lungs, Spleen, Liver, BALF, Peritoneal exudate, P(Flow cyt.), F (bacterial, killing assay) |
| Alim et al. (2020) | Mice (C57BL/6) | 6 wks | M | 78-80 | EDD | *Mtb H37Rv* | 4 × 10^6, 2 × 10^7 CFU | i.v. injection | Lungs, Spleen, Liver, BALF, Peritoneal exudate, P(CBA, Flow cyt.), F (CFU, killing assay) |
| Martens et al. (2007) | Mice (C57BL/6, Ins2Akita, ICR) | ≥ 8 wks | M, F | NR | i.p STZ | *Mtb* Erdman, Con A, anti-CD3 | ~50 CFU | Aerosol | Lungs, Spleen, P(Flow cyt., ELISA), F(CFU, ELISPOT) |
| Cheekatla et al. (2016) | Mice (C57BL/6) | 4-6 wks | F | NR | i.p STZ, NA | *Mtb H37Rv* | 0.5 × 10^6 to 4 × 10^6 CFU/ml | Aerosol | BALF, Lungs, P(Confocal microscopy, IHC, ELISA), T(qpCR), F(lipid assay, mac. infection) |
| Martinez et al. (2016) | Mice (C57BL/6, RAGE-/-) | 8 wks | M | NR | i.p STZ | *Mtb* (*H37Rv,* Erdman), latex beads | MOI 10, 10^6 CFU | Direct infection, Intratracheal | BALF, Peritoneal fluid, Bone marrow, P(Flow cyt., immunoblotting), F, T(qPCR) |
| Tripathi et al. (2019) | Mice (C57BL/6) | 6 wks | F | 50 | i.p STZ, NA | *Mtb H37Rv,* Recombinant IL-22 | 1 × 10^6 to 4 × 10^6 CFU/ml | Aerosol | Lungs, Blood, P(Flow cyt., ELISA, ICS, confocal microscopy), T(qRT-PCR), F(Histology/lung pathology) |
| Sathkumara et al. (2021) | Mice (C57BL/6) | 4-6 wks | M | NR | EDD | *Mtb H37Rv* | 10-20 CFU | Aerosol | Fecal, Lung homogenates, T(rRNA, qPCR), F |
| Oswal et al. (2023) | Mice (C57BL/6) | 4 wks | M, F | 54 | MFD | *Mtb HN878* | 3 × 10^6 CFU/mL | Aerosol | Lung, Serum, Adipose tissue, P(IHC, ELISA, Western blotting), F(Histological) |
| Saiki et al. (1980) | Mice (ICR) | NR | M | NR | i.p STZ | *Mtb* Schacht, *P.aeruginosa* | 1 mg live Mtb, 2 × 10^6 CFU | i.v. Mtb, i.p. injection P.aeruginosa | Peritoneal cells, F(Phagocytosis/killing assay) |
| Arias et al. (2019) | Mice (C3HeB/FeJ) | 6-8 wks | F | 270 | HFD | BCG, *Mtb H37Rv* | 50 CFU | Aerosol | Lungs, Fecal, Cecum, F(CFU, lung pathology), P(Luminex xMAP), T(rRNAseq, microbiota profiling) |
| Sugawara & Mizuno (2008) | Rats | 6 wks | F | NR | Diabetic | *Mtb* Kurono | 3 × 10^6 CFU | Aerosol | Lungs, Spleen, T(RT-PCR, mRNA), F(NO assay) |
| Podell et al. (2014) | Guinea Pigs (Dunkin-Hartley) | NR | NR | 60 | s.c STZ | *Mtb* H37Rv | ~20 bacilli | Aerosol | Lung, Liver, Spleen, Serum, T(RT-PCR), P(Flow cyt., lipid), F(Histopathology, CFU) |
| Podell et al. (2012) | Guinea Pigs | NR | NR | 60 | Sucrose fed | *Mtb* H37Rv | 10^6 CFU/mL | Aerosol | Lung, Serum, Mediastinal LNs, P(ELISA), F(CFU, metabolic) |

.
